# Spatiotemporal patterns and spatial spillover effects of public service supply for national fitness in China

**DOI:** 10.3389/fpubh.2026.1879867

**Published:** 2026-09-09

**Authors:** Haonan Sun, Maoteng Cheng, Pengfei Tai, Fan Jiang, Qiaojing Wang, Fugao Jiang

**Affiliations:** College of Physical Education and Sport Science, Qufu Normal University, Qufu, China

**Keywords:** influencing mechanism, nationwide fitness public service, service supply level, spatial econometric model, spatiotemporal evolution characteristics

## Abstract

Public service for nationwide fitness serves as a key pillar for advancing the Healthy China initiative and reducing health inequities. However, regional development disparities and supply–demand mismatches remain prominent, and empirical research from a public health perspective on the long-term spatiotemporal evolution and spatial spillover effects of such services is still lacking. Using panel data from 30 Chinese provinces from 2013 to 2022, this study measures the supply level of public services for nationwide fitness using the CRITIC-entropy weight method, and employs spatial trend surface analysis, spatial autocorrelation, and a spatial lag model (SAR) to investigate its spatiotemporal patterns and spatial spillover effects. The results show that the supply level of China’s nationwide fitness public services exhibits a three-stage upward trend, with an average annual growth rate of 7.18%, and an overall spatial pattern characterized by higher levels in the east than in the west, and higher in the south than in the north. The disparity between the eastern and western regions has narrowed, whereas the north–south gap has continued to widen. Economic development level and urbanization level demonstrate bidirectional positive spatial spillover effects; population density, government support, and educational structure only affect local supply levels; transportation accessibility shows no significant effect. Therefore, differentiated regional policies and strengthened interprovincial cooperation are needed to optimize resource allocation with a focus on health equity. These findings provide a reference for formulating public health strategies, alleviating health inequities, and improving the overall health status of residents.

## 1 Introduction

Public service for nationwide fitness is a core component of health promotion and a key instrument for advancing population health and health equity in China. From the perspective of the social determinants of health, whether individuals can equitably access fitness facilities, professional guidance, and health monitoring services directly affects their physical activity levels, chronic disease risks, and overall health status. The World Health Organization has pointed out that where we are born, grow, live, work and age, and our access to power, money and resources influence our health outcomes more than genetic influences or healthcare. Geographic variations in the level of public service provision are a significant manifestation of such disparities. Spatial justice theory further emphasizes that the supply levels of public resources should equitably meet the health needs of populations across different regions, rather than being based solely on economic efficiency or administrative convenience. Thus, public fitness services are not merely a matter of sports policy but also a spatial justice issue with profound public health implications.

As a key pillar of the Healthy China strategy, public fitness services aim to reduce disparities in physical activity and health outcomes across different social groups. In 2024, the Third Plenary Session of the 20th Central Committee of the Communist Party of China adopted the “Resolution of the Central Committee of the Communist Party of China on Further Deepening Reform Comprehensively to Advance Chinese Modernization,” which proposed to “improve the public service system for nationwide fitness” (1), providing scientific guidance for the development of nationwide fitness public services. Subsequently, the Mass Sports Department of the General Administration of Sport of China issued the “2026 Key Points for Mass Sports Work,” which further emphasized the need to “establish a long-term and effective supply mechanism for basic public services for nationwide fitness” (2). Guided by these policies, China’s nationwide fitness public services have achieved notable progress in supply scale, including infrastructure development and service network coverage. However, challenges such as uneven regional development, unequal service distribution, and insufficient supply–demand matching remain prominent (3). From a health equity perspective, such imbalances may further widen regional health gaps and undermine the goal of equitable population health, as limited access to fitness resources reduces physical activity participation among residents in disadvantaged regions, thereby increasing their risk of chronic diseases and the intergenerational transmission of health disadvantages.

From a spatial perspective, regional public services do not exist in isolation. Public policy diffusion, resource mobility, and inter-regional cooperation can generate spatial spillover effects. Specifically, in terms of policy diffusion, successful policy experiences from neighboring provinces—such as government service procurement models and smart sports platform development—are readily learned and imitated by local governments. In terms of resource mobility, capital, talent, and sports event resources can flow across provinces, benefiting surrounding areas. In terms of regional competition, local governments engage in yardstick competition in the public service sector, whereby improvements in the supply levels of neighboring provinces pressure local governments to follow suit. However, how these mechanisms specifically operate in the domain of public fitness service supply, and the extent to which such spillover effects are transmitted through channels such as economic linkages, geographic proximity, or administrative imitation, remain lacking in systematic theoretical explanation and empirical testing in the existing literature. In particular, the potential implications of these spatial spillover effects for inter-regional health equity—for instance, whether improvements in service levels in developed regions benefit less developed areas through spillovers (diffusion effects) or exacerbate disparities through suction effects (backwash effects)—have not received sufficient attention. Internationally, similar studies have been conducted in developed countries in Europe and North America; for example, researchers have examined the impact of the spatial distribution of sports infrastructure on regional health disparities (4). However, whether the conclusions of these studies are applicable to China, which is undergoing rapid urbanization, remains to be empirically tested.

To address the above research gaps, this study adopts the dual perspective of the social determinants of health and spatial justice, utilizes panel data from 30 Chinese provinces spanning 2013 to 2022, measures the supply level of public fitness services, and examines their spatiotemporal evolution and spatial spillover effects. This study makes exploratory contributions to the existing literature in the following aspects. At the theoretical level, it introduces the social determinants of health and spatial justice theories into the field of national fitness research, constructs a theoretical framework for analyzing the relationship between the spatial allocation of fitness services and health equity, and identifies three transmission channels of spatial spillovers. At the methodological level, it applies spatial econometric models to long-term provincial panel data to test the existence and direction of cross-regional spillover effects, thereby supplementing the application of this method in this field. At the practical level, based on empirical findings, it proposes policy recommendations for optimizing resource allocation and narrowing regional disparities, aiming to provide a reference for advancing health equity goals under the Healthy China strategy.

## 2 Literature review and theoretical foundations

### 2.1 Literature review

The supply level of national fitness public services is a core indicator for measuring the effectiveness of public health modernization, which comprehensively reflects the regional supply capacity and service quality of public sports services, including fitness venue construction, activity organization, scientific fitness guidance, and sports atmosphere cultivation. With the continuous deepening of the national fitness strategy, the Chinese government has successively introduced a number of supporting policies to improve the public sports service system. Consequently, issues of regional disparities, spatial patterns, and equity in development have become core research hotspots in the fields of public sports and public health. Existing research focuses primarily on four major directions: the construction of evaluation systems, spatial patterns and regional disparities, influencing factors and policy effects, and digital technology innovation and application. In terms of evaluation system construction and quantitative measurement, existing research has moved beyond the limitations of single-indicator evaluations, establishing multi-dimensional assessment systems that balance supply scale and service quality (5, 6). However, notable differences exist among studies in indicator selection and weighting methods: some emphasize objective facility indicators, others prioritize subjective satisfaction evaluations; some employ the entropy weight method, while others use the analytic hierarchy process. This methodological divergence limits the comparability of research findings, reflecting that the field has not yet established a unified evaluation standard. Regarding spatial patterns and regional disparities, studies have confirmed that the supply of public fitness services in China exhibits significant spatial imbalances. From a micro urban perspective, fitness resources exhibit a core-agglomeration pattern, with insufficient supply in suburban areas (7). From a macro provincial perspective, the supply in the eastern region is significantly better than that in the central and western regions (8). Notably, the existing findings are contradictory: some studies show that regional disparities continue to widen, while others observe a trend toward convergence. This divergence may arise from differences in the choice of study periods, but few studies have explored the underlying mechanisms in depth. In terms of influencing factors and policy effects, existing research confirms that government financial investment is the core driving force, with factors such as economic development level, population structure, and policy support intensity all exerting significant effects (9–12). Studies using symbiosis models have confirmed a bidirectional interactive relationship between public fitness services and the development of the sports industry (13). However, most of these studies employ traditional panel regression models that assume regions are independent of one another, failing to account for spatial spillover effects, which may lead to estimation bias. In terms of digital innovation and smart technology applications, the use of digital platforms and intelligent algorithms can effectively address issues such as fragmented traditional service resources and insufficient supply precision (14, 15), while smart city information frameworks have enabled precise allocation and intelligent delivery of fitness resources (16). Personalized recommendation models based on neural network algorithms can provide differentiated fitness services, improving resource utilization efficiency and service inclusiveness (17). These studies provide technical references for the subsequent formulation of optimization strategies in this research.

Compared with international research, studies in China share both commonalities and differences in perspective and methodology. At the international level, scholars have extensively examined the impact of the spatial distribution of sports infrastructure on public health, yet these studies exhibit notable disagreements in their conclusions and methodologies. Gordon-Larsen et al. found that low-income and minority communities in the United States had significantly lower accessibility to sports facilities, which directly contributed to health disparities in physical activity and obesity rates (18). However, this study only used data from 1994 to 1995 and was essentially cross-sectional in nature. Downward et al. (4) demonstrated in their study on England that the accessibility of sports facilities is significantly correlated with the degree of regional deprivation, thereby affecting sports participation levels and health inequality across different populations. Regarding the territorial allocation and equitable access to public resources, scholars have adopted broader perspectives to examine the impact of spatial configuration on health and sustainable development. Dincă et al. (19), in a comparative study of six European member states, found a spatial dependency between macroeconomic indicators and the financing performance of health systems, suggesting that the allocative efficiency of public resources is not independent of inter-regional economic linkages. Pricope et al. (20), in their study on EU SDG 11 (Sustainable Cities and Communities), employed principal component analysis to construct a sustainable development index, revealing spatial heterogeneity in the process of urban sustainable development. That study emphasized that equitable access to public infrastructure and services is a core dimension for measuring progress in sustainable development, and that differences in resource allocation across regions directly affect the degree to which sustainable development goals are achieved. Yang et al. (21), in a study of 30 developing Asian countries, found that carbon emissions significantly reduced life expectancy, while the adoption of renewable energy weakened this negative association. Collectively, these studies highlight the importance of employing spatial methods to analyze the equity of public services, thereby providing methodological references for this research.

In summary, the aforementioned studies provide methodological references for this research; however, significant research gaps remain to be addressed. First, most existing studies employ traditional panel regression models that assume regions are independent of one another, failing to account for the spatial spillover attributes of public fitness services. In fact, provincial public fitness service supply does not exist in isolation; local service development can generate significant spatial radiation and spillover effects on neighboring regions through pathways such as policy diffusion, resource mobility, and regional competition. Ignoring this spatial dependence may lead to model misspecification and biased estimation results, thereby failing to reveal cross-regional interaction mechanisms. Second, there is a lack of systematic empirical research on the spatial spillover mechanisms of public fitness services specifically in China. Existing studies generally treat spatial spillover effects as a monolithic phenomenon, without further distinguishing the differentiated roles of various influencing factors between local and neighboring regions. To address these gaps, this study constructs a comprehensive evaluation system for the supply of public fitness services, employs the CRITIC method for objective weighting, uses spatial econometric models to analyze spatiotemporal evolution characteristics, and empirically tests spatial spillover effects and their underlying mechanisms through the spatial autoregressive (SAR) model.

### 2.2 Theoretical foundations

#### 2.2.1 Social determinants of health theory

The social determinants of health theory posits that the primary roots of health inequities lie not in differences in medical services, but in the unequal conditions in which people are “born, grow, live, work, and age.” These conditions include income level, educational attainment, living environment, occupational exposure, social support networks, and access to community resources. The supply level of public fitness services, as a health-supportive environment, directly affects the physical activity opportunities of residents in different regions through its spatial distribution. From a theoretical mechanism perspective, the supply of fitness services affects health equity through three pathways: a behavioral pathway, whereby accessible fitness facilities reduce the time–space costs of physical activity and promote higher levels of physical activity; a preventive pathway, whereby regular physical activity helps lower the risk of chronic diseases such as cardiovascular disease, diabetes, and obesity; and a cumulative pathway, whereby the absence of a health-supportive environment interacts with other health risk factors such as low income and low education to create intergenerational transmission of health disadvantage. From this perspective, regional differences in the supply level of fitness services are essentially a manifestation of territorial inequality in the social determinants of health. This study adopts the social determinants of health theory as the theoretical basis for variable selection—core variables such as economic development level, population density, government support, educational structure, transportation accessibility, and urbanization level are precisely the specific manifestations of the social determinants of health emphasized by this theory.

#### 2.2.2 Spatial justice theory

Spatial justice theory emphasizes that the spatial allocation of public resources should equitably meet the well-being needs of populations in different regions, rather than being subordinated solely to economic efficiency or administrative convenience. The core critique of this theory is that, under the dominance of capital logic, public resources tend to flow toward economically developed and politically influential regions, while disadvantaged regions are systematically marginalized. In the domain of public services, spatial injustice manifests in three specific forms: allocative injustice, whereby resources are excessively concentrated in developed regions; accessibility injustice, whereby residents of less-developed regions face higher costs and lower quality in accessing services; and participatory injustice, whereby disadvantaged groups are excluded from resource allocation decisions. More critically, spatial spillover effects can move in two opposing directions, with diametrically opposed implications for justice. Diffusion effects refer to resource flows and policy diffusion from developed regions benefiting neighboring less-developed regions, helping to narrow regional disparities and aligning with the ideal of spatial justice; backwash effects refer to developed regions drawing in resources from surrounding areas, with their development occurring at the expense of neighboring regions, thereby violating the principle of spatial justice. This study will use spatial econometric models to test the direction of spatial spillover effects in the supply of public fitness services in China, thereby determining whether its spatial pattern approaches or deviates from the ideal state of spatial justice.

The two theories complement and reinforce each other in this study. The social determinants of health theory answers how inequality in the spatial distribution of fitness service supply translates into inequality in health outcomes through the behavioral, preventive, and cumulative pathways; spatial justice theory provides the normative criteria for judging whether the spatial allocation is fair. The combination of the two enables this study both to explain the current situation and to make value judgments.

## 3 Research design

### 3.1 Construction of the evaluation indicator system

Based on a systematic review of relevant policy documents and academic literature, and adhering to the principles of data availability, systemic coherence, and feasibility, this study draws upon prior research (22–24) to select five first-level indicators: financial security, fitness guidance, organizational management, venue facilities, and physical health monitoring. A comprehensive evaluation framework comprising eight quantifiable second-level indicators was subsequently constructed, as presented in Table 1. This indicator system covers both input-type and output-type indicators, balancing the scale and quality of service supply, and can comprehensively reflect the supply capacity of public fitness services across provinces. Specifically, financial security is represented by two indicators: per capita public expenditure on mass sports and per capita expenditure from the welfare fund of the national fitness lottery. Financial investment serves as a fundamental guarantee for narrowing the gaps in the supply of public fitness services between regions and among different income groups; rational allocation of public funds helps address the shortcomings in service supply for vulnerable groups such as rural and low-income communities under market mechanisms, thereby alleviating health inequalities arising from economic disparities. Fitness guidance is measured by the number of social sports instructors. Their quantity and spatial distribution directly affect the prevalence of scientific fitness behaviors among residents; increasing the supply of social sports instructors not only helps enhance the enthusiasm and effectiveness of public participation in physical exercise but also reduces health disparities caused by a lack of professional guidance or information. Organizational management is reflected by two indicators: the number of employees in sports administrative agencies and the number of sports social organizations. A sound administrative and social organization system is key to enhancing the accessibility and coverage of public fitness services; efficient organizational management capacity can extend service resources more equitably to marginalized groups, preventing excessive concentration of resources in advantaged areas or populations, thereby promoting health equity. Sports facilities are measured by the per capita area of sports venues as the core indicator. As the material foundation for residents’ participation in physical exercise, increasing the per capita area of sports venues—especially through targeted investment in resource-deficient communities—can significantly enhance public service supply capacity and effectively narrow the health opportunity gap caused by differences in living environments. Physical health monitoring is jointly represented by two indicators: the number of national physical fitness monitoring stations (sites) and the annual number of people receiving national physical fitness tests. The coverage breadth and service scale of this system reflect the capacity to identify, assess, and intervene in the health status of residents; expanding monitoring coverage helps to promptly detect and precisely address various health shortcomings, thereby curbing the intergenerational accumulation and structural entrenchment of health inequalities.

**Table 1:** Evaluation indicator system for the supply level of public services for national fitness.

First-level indicator	Second-level indicator	Unit	Indicator attribute	Weight
Financial security	Per capita fiscal expenditure on mass sports	10,000 CNY	+	0.1305
	Per capita public welfare lottery fund expenditure on national fitness	10,000 CNY	+	0.1509
Fitness guidance	Number of social sports instructors	Persons	+	0.1036
Organizational management	Number of employees in sports administrative agencies	Persons	+	0.1307
	Number of sports social organizations	Units	+	0.1125
Venue facilities	Per capita sports venue area	m^2^	+	0.1480
Physical health monitoring	Number of national physical fitness monitoring stations (sites)	Units	+	0.0957
	Annual number of individuals undergoing national physical fitness testing	Persons	+	0.1281

### 3.2 Research methods

#### 3.2.1 CRITIC method

Within a multi-indicator comprehensive evaluation framework, the CRITIC weighting method achieves a more objective weight assignment by comprehensively examining the information content, internal contrast intensity, and conflict degree of the indicators. Compared to the entropy weight method or the standard deviation method, which focus solely on data volatility, the advantage of this approach lies in its integration of correlation analysis among indicators with the assessment of their dispersion, thereby enhancing the rationality of weight allocation. This study employs the CRITIC method to determine the specific weights of each dimension of public services for national fitness and subsequently constructs a composite supply level evaluation index. The detailed calculation procedure is as follows:

First, data standardization is performed. Suppose there are *n* evaluation indicators and *m* objects to be evaluated, forming a matrix *X = (x_ij_)m × n*.


$${X_{\mathrm{ij}}}^{\prime }=\frac{x_{\mathrm{ij}}-\min x_{\mathrm{ij}}}{\max x_{\mathrm{ij}}-\min x_{\mathrm{ij}}}$$

Where *x_ij_* denotes the original value of the *j*-th indicator for the *i*-th object, and *x’_ij_* represents the corresponding standardized value.

Second, the information content of each indicator is calculated.


$$I_{j}=d_{j}c_{j}=\sqrt{\frac{\sum_{i=1}^{m}{\left(x_{\mathrm{ij}}^{\prime }-{\bar{x}}_{j}^{\prime }\right)}^{2}}{m-1}\sum_{i=1}^{m}(1-r_{\mathrm{ij}})}$$

Where *I_j_*, *d_j_* and *c_j_* denote the information content, contrast intensity, and conflict degree of the *j*-th indicator, respectively, and *r_ij_* represents the correlation coefficient between indicator *i* and indicator *j*.

Finally, the weights and the supply level evaluation index are calculated.


$$T_{i}=\sum_{j=1}^{n}w_{j}x_{\mathrm{ij}}^{'}=\sum_{j=1}^{n}\frac{I_{j}}{\sum_{j=1}^{n}I_{j}}x_{\mathrm{ij}}^{'}$$

Where *w_j_* represents the weight of the *j*-th indicator, and *T_i_* denotes the supply level evaluation index for province *i*.

#### 3.2.2 Global trend surface analysis

Trend surface analysis is a technique based on mathematical surface modeling that fits discrete sampling point data in two-dimensional space into a continuous, three-dimensional visual surface, thereby effectively simulating the spatial distribution patterns and dynamic evolution of geographic phenomena. Employing the national fitness supply level evaluation index as the data source, this study applies this method to dissect its spatial differentiation patterns and reveal its dynamic evolutionary characteristics on a global scale.

#### 3.2.3 Spatial autocorrelation analysis

To further investigate the intrinsic spatial structure of the supply level of public services for national fitness, this study employs a spatial autocorrelation analysis approach. By measuring the spatial dependence among geographic units, this method identifies the clustering or dispersion patterns of the supply level, thereby revealing its overall distribution pattern from a spatial dimension.

#### 3.2.4 Spatial lag model

According to the first law of geography, the attribute characteristics of geographic units generally exhibit spatial dependence, meaning that the attribute value of a given unit is correlated with the same characteristic of its neighboring units, with the strength of correlation typically intensifying as distance decreases (25). As conventional regression models struggle to adequately capture such spatial correlation effects, this study employs the spatial lag model (SAR) from spatial econometrics and incorporates the Lee–Yu correction into the model estimation, to examine the spatial interaction effects of the supply level of public services for national fitness across provincial-level units, and to identify the spatial effects generated by relevant influencing factors. The model specification is as follows:


$$y_{\mathrm{it}}=\lambda w_{i}^{\prime }Y_{\mathrm{it}}+x_{\mathrm{it}}^{\prime }\beta +\mu_{i}+\gamma_{t}+\varepsilon_{\mathrm{it}}$$

Where *λ* denotes the spatial coefficient, *w’* represents the spatial weight matrix, *y_it_* is the dependent variable for province *i* in year *t*, *x’_it_* is the vector of independent variables for province *i* in year *t*, *β* is the regression coefficient, *μ_i_* and *γ_i_* denote the individual and time fixed effects, respectively, and *ε_it_* is the random disturbance term.

### 3.3 Selection of influencing factors

Economic development constitutes a fundamental prerequisite for enhancing the supply capacity of public fitness services for the entire population (26). A sound economic environment bolsters local fiscal capacity and household consumption, thereby enabling greater public investment in sports, the improvement of fitness infrastructure, and the enrichment of public fitness service provision. Simultaneously, it promotes the marketization and diversification of the sports industry and optimizes the regional allocation of sports resources, ensuring sustained, high-quality public fitness service delivery. In this regard, economic development serves as an essential precondition for narrowing regional disparities in fitness services and advancing health equity. Population density is a crucial spatial factor influencing the efficiency and modes of public fitness service provision (27). Population agglomeration facilitates the intensive use of public fitness resources, reduces per capita supply costs, and enhances the coverage and utilization efficiency of fitness facilities. Moreover, the large-scale demand for fitness arising from dense populations can stimulate sports market vitality, attract private capital into service development, foster innovative fitness service formats, and drive the continuous optimization of the public service system. This, in turn, contributes to the precise matching of supply and demand for fitness services and promotes health equity across all regions. Government support functions as the core driving force behind the effective supply of public fitness services (28). Coordinated planning, policy support, and fiscal investment by the government directly determine the overall quality of public fitness facility construction, sports activity organization, and service operation. Guided by policy frameworks and underpinned by financial safeguards, the efficiency of public sports resource allocation can be substantially improved. Furthermore, through a range of measures—including government procurement of services and tax incentives—social actors can be mobilized to participate in service delivery, thereby shaping a government-led, multi-stakeholder collaborative supply structure that advances the equalization and inclusiveness of public services. The educational composition of the population profoundly shapes the developmental trajectory and quality of public fitness services by influencing residents’ health awareness and the hierarchy of their fitness needs (29). In regions with higher levels of educational attainment, residents tend to exhibit greater health literacy and stronger fitness consciousness, along with higher demand for premium and refined fitness services. This demand-side upgrading exerts pressure on local governments to optimize service delivery models and enhance service content, thereby accelerating the transition toward specialized, high-quality public services and consolidating the livelihood foundation of health equity. Transportation accessibility constitutes a fundamental condition constraining the real-world effectiveness of public fitness service delivery (30) and is intimately linked to health equity. A well-developed transportation network reduces the time–space distance of residents’ fitness-related travel, expands the service radius of fitness facilities, and lowers the cost of public participation in fitness activities. This effectively enhances the utilization efficiency of public services, mitigates disparities in service accessibility between urban and rural areas and across regions, and facilitates the inclusive sharing of fitness services. The level of urban development acts as both a critical vehicle and a core driving factor for the high-quality development of public fitness services (31). The urbanization process, characterized by population concentration, infrastructure improvement, and the upgrading of grassroots governance, supports the scaled and centralized provision of public fitness services. At the same time, urbanization facilitates the integration of public sports services into the coordinated urban–rural development framework, promotes the downward flow of high-quality resources to community and rural levels, and helps resolve the problem of resource imbalances between urban and rural areas. This contributes to the establishment of a region-wide, inclusive, and shared fitness service network, thereby fostering balanced regional development of fitness services.

In summary, this study identifies six indicators as key factors influencing the supply level of public fitness services: economic development, population density, government support, educational composition of the population, transportation accessibility, and urbanization level (Table 2). Adopting the perspective of health equity, this research employs spatial econometric methods to systematically investigate the spatiotemporal evolution characteristics, spatial dependence, and spatial spillover effects of public fitness service supply in China, thereby offering an in-depth analysis of the functional mechanisms of these factors and their regional interactions.

**Table 2:** Selection of influencing factors for the supply level of public services for national fitness.

Influencing factor	Indicator
Economic development level (x1)	The ratio of gross regional product to the year-end permanent resident population.
Population distribution density (x2)	Population per unit land area
Government support intensity (x3)	Proportion of mass sports expenditure in public sports fiscal spending
Population educational structure (x4)	Number of higher education enrollments per 100,000 population
Transportation convenience (x5)	Transportation network density
Urbanization development level (x6)	Regional urbanization rate

### 3.4 Data sources

This study employs panel data spanning from 2013 to 2022 for 30 Chinese provinces and municipalities as the research sample; Tibet is excluded due to missing data. The foundational data for the evaluation indicator system of the supply level of public services for national fitness were sourced from the China Statistical Yearbook and the China Sports Statistical Yearbook. Data on influencing factors were obtained from the China Statistical Yearbook, the China Sports Statistical Yearbook, and the National Bureau of Statistics. To ensure the integrity of the dataset, a combination of interpolation and imputation methods was applied to address individual missing data points. In the data of the evaluation index system for the supply level of public fitness services, three indicators—namely, the number of national physical fitness monitoring stations (sites), the annual number of individuals receiving physical fitness tests, and the number of sports social organizations—had missing data in 2019. Additionally, the number of social sports instructors was missing in 11 provinces including Tianjin and Inner Mongolia in 2013; the number of national physical fitness monitoring stations (sites) was missing in Xinjiang in 2015; and the annual number of individuals receiving physical fitness tests was missing in Qinghai in 2013 and in Xinjiang in 2015. All of the above missing values were filled using the linear interpolation method. For certain indicators in specific provinces and individual years, the observed values exhibited sharp fluctuations relative to the preceding or following year, which were suspected to result from abnormal data entry formats; after their removal, the missing values were also filled using the linear interpolation method. The data for the influencing factor variables had no missing values.

## 4 Spatiotemporal evolution characteristics of the supply level of public services for national fitness

### 4.1 Temporal evolution characteristics

#### 4.1.1 Overall evolutionary trend

**Figure 1:**
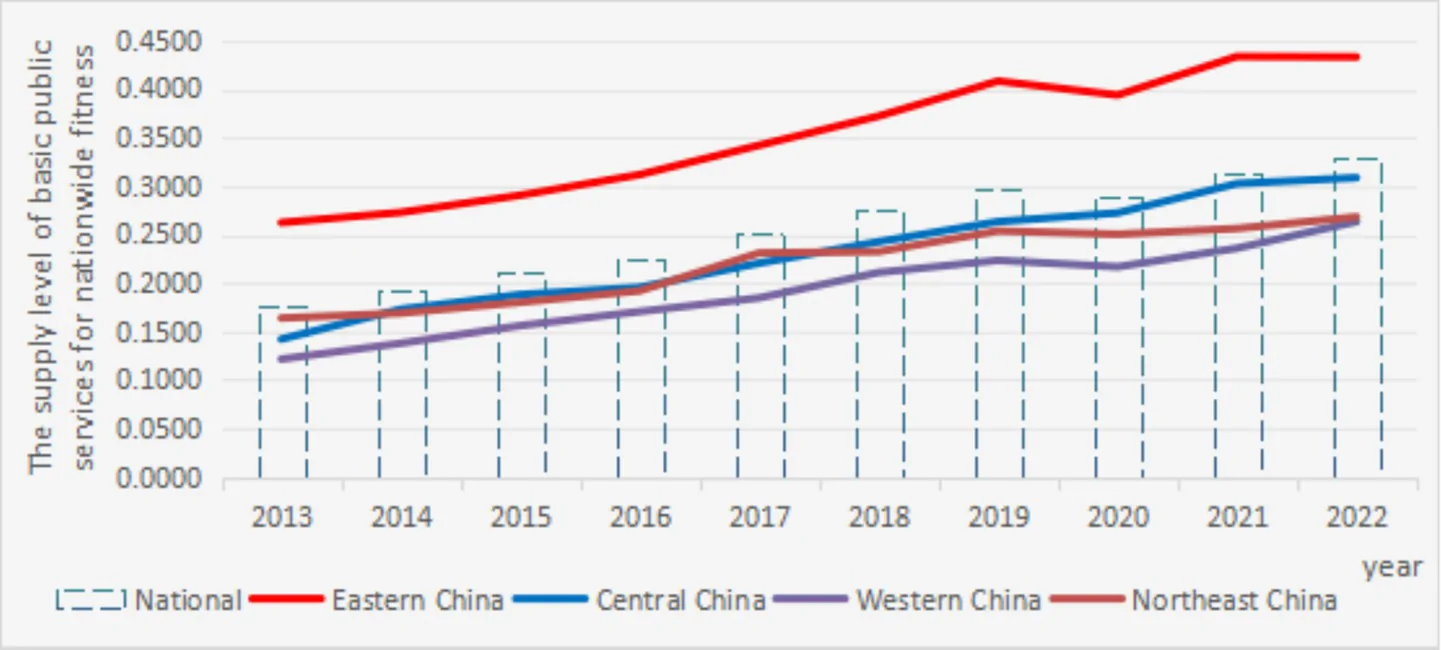
Trends in the supply level of public services for national fitness at the national level and across the four major regions.

Figure 1 presents the measurement results based on the CRITIC objective weighting method, revealing distinct temporal evolutionary characteristics in the supply level of public services for national fitness across 30 Chinese provinces and municipalities from 2013 to 2022. The overall supply level maintained an upward trajectory, with the composite evaluation index rising from 0.1768 in 2013 to 0.3299 in 2022, reflecting an average annual growth rate of 7.18%. This sustained growth pattern reflects the remarkable effectiveness of the nation’s continuous investment and policy guidance in developing the public service system for national fitness. Moreover, from a public service perspective, it demonstrates that the strategic initiatives promoting digital transformation, accelerating the construction of a leading sporting nation, and fostering the sports industry are gradually being implemented and yielding tangible results.

Regarding the evolutionary trajectory, the development of the supply level of public services for national fitness in China during the study period exhibited a three-stage pattern characterized by “rapid expansion, dynamic adjustment, and resilient qualitative improvement”: Rapid expansion stage (2013–2017): The national supply level surged from 0.1768 to 0.2501, with an average annual growth rate of 9.06%. The core mission of this stage was to establish services “from scratch,” with development primarily driven by strong policy impetus and large-scale infrastructure investment, thereby initially constructing a nationwide backbone network for the supply of public services for national fitness. Dynamic adjustment stage (2018–2020): The national index fluctuated upward from 0.2747 to 0.2898, with the average annual growth rate decelerating to 2.71%. During this stage, the focus shifted from scale expansion to connotative enhancement, as the system entered a phase of absorbing and consolidating prior developmental achievements. Under the impact of the pandemic, the shortcomings of existing service models were exposed, propelling the sector into an exploratory period of service transformation, wherein the development logic gradually shifted from “prioritizing quantity” to “improving quality.” Resilient qualitative improvement stage (2021–2022): The national level increased from 0.3143 to 0.3299, with the average annual growth rate rebounding to 4.96%. The system demonstrated considerable resilience in coping with external shocks, and the developmental momentum progressively shifted from factor input-driven growth to service innovation and digital empowerment. This transition signifies that the public service system for national fitness has entered a new stage of high-quality development characterized by a more optimized structure, higher efficiency, and greater sustainability. Notably, during the period 2020–2022, although the COVID-19 pandemic significantly impacted population mobility and the opening of public venues, the supply level continued to grow positively, reflecting that the rapid popularization of digital fitness services (e.g., online guidance, cloud-based events) offset the adverse effects of the pandemic to some extent. The impact of the pandemic on regional disparities presents a complex dual effect. On the one hand, the rise of online fitness services in the early stage of the pandemic reduced the constraints of geographical distance on service access, allowing residents in remote central and western regions to obtain high-quality fitness resources that were previously difficult to reach, which in theory helps narrow regional disparities. On the other hand, as the pandemic persisted, a differentiation effect gradually emerged: developed eastern regions, with their well-established digital infrastructure and higher levels of digital literacy among residents, were able to complete the transition from offline to online service delivery more quickly; in contrast, some central and western provinces, with weaker digital infrastructure and a more pronounced digital divide among older adults, faced greater losses in service accessibility under the pandemic shock. Due to data availability limitations, this study was unable to precisely quantify the digital service transformation triggered by the pandemic or its impact on regional disparities. However, the above mechanistic analysis suggests that the pandemic may have exacerbated the “digital divide” in access to public fitness services in the short term. It should be noted that the above three-phase division is primarily based on descriptive observations of the average annual growth rates, and is intended to facilitate the presentation of the temporal variation characteristics of the supply level.

#### 4.1.2 Regional disparity dynamics

From a regional perspective, the supply level of public services for national fitness across China’s four major regions exhibited an overall upward trend from 2013 to 2022; however, significant disparities were evident in both the pace and magnitude of improvement. The eastern region consistently maintained a leading position, with its index value rising from 0.2622 in 2013 to 0.4330 in 2022, at an average annual growth rate of 5.73%, reflecting a pronounced advantage. The central region experienced the most rapid growth, increasing from 0.1421 to 0.3085 at an average annual rate of 9.00%, indicating that the supply capacity of public sports services has been significantly enhanced under the impetus of national strategies such as the Rise of Central China. The western region started from a relatively low baseline (0.1215 in 2013) and reached 0.2632 in 2022, with an average annual growth of 8.97%, reflecting the positive outcomes of the Western Development Strategy in promoting the equalization of basic public services. The northeastern region experienced relatively moderate growth, rising from 0.1638 to 0.2681 at an average annual rate of approximately 5.63%, with its overall level situated between that of the central and western regions.

Overall, the supply level across the four major regions exhibits a gradient pattern of eastern region > central region > northeastern region > western region. From a health equity perspective, this gradient implies that residents in the central, western, and northeastern regions still lag behind those in the eastern region in terms of access to fitness-supportive environments, which may contribute to persistent regional disparities in physical activity levels and chronic disease risks.

### 4.2 Spatial trend surface analysis

**Figure 2:**
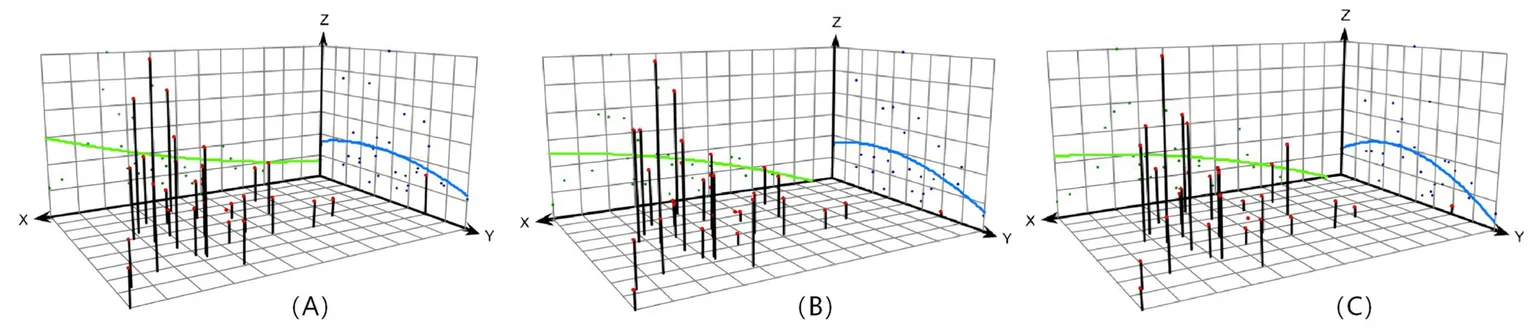
Spatial trend surface of the supply level of basic public services for nationwide fitness. Subfigures **(A–C)** correspond to the research years of 2013, 2018 and 2022 respectively.

Figure 2 presents the three-dimensional spatial trend surface visualizations of the supply level of public services for national fitness in China for the years 2013, 2018, and 2022. In these visualizations, the *Z*-axis denotes the supply level, with the calculated values for each province represented by black clustered columns; the *X*-axis and *Y*-axis correspond to the due east and due north directions, respectively. The green curve represents the trend line along the west–east direction, while the blue curve depicts the trend line along the south–north direction; (*x_i,_ y_i_*) and *Z_i_*(*x_i,_ y_i_*) denote the spatial coordinates and the trend function value of province *i*, respectively. This three-dimensional spatial representation intuitively illustrates the regional disparities in the supply level and its spatiotemporal evolution characteristics.

From the perspective of geographical distribution patterns, the self-organizing capacity and spatial adaptability of the supply level from 2013 to 2022 resulted in a pronounced characteristic of “stronger in the east, weaker in the west” and “higher in the south, lower in the north.” Along the west–east transect, the supply level exhibited a continuously rising trend as longitude shifts eastward, indicating that the eastern region generally outperforms the western region. Along the south–north transect, the trend line largely displayed an inverted U-shaped distribution, with the supply level in the southern region being relatively higher than that in the northern region.

From the perspective of temporal evolution, the gradually decreasing slope of the green trend line along the west–east direction indicates that the supply level in the western region is progressively catching up with that of the east, narrowing the regional disparity. This convergence is primarily attributable to three mechanisms: first, the continuous increase in central government fiscal transfers has significantly improved the financial accessibility of national fitness initiatives in western provinces; second, regional coordinated development strategies such as the Western Development Program have facilitated a westward-oriented allocation of sports infrastructure; third, paired assistance mechanisms have enabled the transfer of advanced practices and experiences from the eastern region to the western region. This implies that residents in less-developed regions are gaining improved access to fitness-supportive environments, helping to reduce geographically determined health inequalities. In contrast, the increasing slope of the blue trend line along the south–north direction in its ascending segment indicates that the supply level in the southern region is growing more vigorously, thereby further widening the gap with the northern region. The widening north–south gap thus signals an emerging health equity risk. The relatively slower growth in the supply level of the northern region may be attributed to several factors: the greater pressure of economic transformation in traditional heavy industrial areas; population outflows, particularly of young laborers, which create a negative cycle of shrinking service demand and declining supply capacity; and the natural constraints imposed by cold winter weather on outdoor fitness activities. From a health equity perspective, this implies that residents of some northern provinces may face higher barriers to accessing fitness resources, which could adversely affect their physical activity levels and chronic disease risk profiles. This finding suggests to policymakers that the traditional “east–west” dichotomy is no longer sufficient to capture the pattern of regional health inequality in China, and that “north–south differentiation” may emerge as a new health equity challenge.

### 4.3 Spatial distribution characteristics

The supply level of public services for national fitness in China currently exhibits a gradient distribution pattern of eastern region > central region > northeastern region > western region. A cross-sectional comparison was conducted across three temporal snapshots—2013, 2018, and 2022—and based on the supply index of all provinces from 2013 to 2022, the quartile method was applied to classify the supply level into four tiers: low-supply provinces (0.0529–0.1710), relatively low-supply provinces (0.1710–0.2278), relatively high-supply provinces (0.2278–0.3113), and high-supply provinces (0.3113–0.7229), in order to systematically identify the differential characteristics in public service supply across provinces and municipalities.

As shown in Table 3, the spatial pattern of the supply level of public services for national fitness in China exhibits dual characteristics: statically, the overall distribution displays a “high in the east, low in the west” pattern, which aligns closely with regional economic development levels and manifests as a stepwise decline from east to west; dynamically, the high-supply provinces demonstrate a diffusion trend from the eastern region toward the central and western regions. Specifically, the high-supply areas were predominantly concentrated in the eastern coastal provinces in 2013, had extended to some central provinces by 2018, and further spread to the central, western, and northeastern regions by 2022. This dynamic evolution of the spatial pattern is closely associated with a series of national strategies and top-level designs that have been continuously advanced in recent years. Following the successive promulgation and implementation of policy documents such as the 13th Five-Year Plan, the “Healthy China 2030” Blueprint, and the Outline for Building a Leading Sporting Nation, the central government significantly increased financial support for basic public services in the central, western, and northeastern regions. Through diversified measures such as increasing general transfer payments, establishing special subsidy funds, and promoting horizontal ecological compensation mechanisms, efforts were intensified to optimize inter-regional resource allocation, thereby effectively improving public service facility conditions in less-developed areas. Concurrently, as the “Internet Plus Government Services” initiative extended into the sports and health sector, digital technologies effectively overcame geographical constraints on public service distribution, enabling the central and western regions to rapidly enhance the accessibility and operational quality of national fitness services through new service models such as remote guidance and smart fitness platforms. Throughout this process, the continuous deepening of regional development strategies, including the Rise of Central China, the Western Development Strategy, and the Comprehensive Revitalization of Northeast China, has provided a solid institutional safeguard and developmental momentum for the balanced advancement of public services for national fitness.

**Table 3:** Spatial distribution of the supply level of public services for national fitness.

Year	Low-supply provinces	Relatively low-supply provinces	Relatively high-supply provinces	High-supply provinces
2013	Tianjin, Hebei, Shanxi, Inner Mongolia, Jilin, Heilongjiang, Anhui, Jiangxi, Hubei, Hunan, Guangxi, Hainan, Chongqing, Guizhou, Yunnan, Shaanxi, Gansu, Qinghai, Ningxia, Xinjiang	Liaoning, Shanghai, Fujian, Henan, Sichuan	Beijing, Shandong	Jiangsu, Zhejiang, Guangdong
2018	Shanxi, Chongqing, Guizhou, Xinjiang	Tianjin, Heilongjiang, Anhui, Jiangxi, Guangxi, Gansu, Qinghai	Hebei, Inner Mongolia, Liaoning, Jilin, Fujian, Hubei, Hunan, Hainan, Sichuan, Yunnan, Shaanxi, Ningxia	Beijing, Shanghai, Jiangsu, Zhejiang, Shandong, Henan, Guangdong
2022		Shanxi, Chongqing, Guizhou, Qinghai, Ningxia, Xinjiang	Tianjin, Inner Mongolia, Jilin, Heilongjiang, Jiangxi, Guangxi, Hainan, Yunnan, Shaanxi, Gansu	Beijing, Hebei, Liaoning, Shanghai, Jiangsu, Zhejiang, Anhui, Fujian, Shandong, Henan, Hubei, Hunan, Guangdong, Sichuan

A comparison of typical provinces reveals that high-supply provinces (e.g., Jiangsu and Zhejiang) share common characteristics: a strong economic foundation, robust fiscal self-sufficiency, a developed sports industry, and an early start in digital infrastructure construction. In contrast, low-supply provinces (e.g., Qinghai, Ningxia, and Xinjiang) generally face structural constraints, including a weak economic base, high fiscal dependence, an excessively large service radius due to sparse population distribution, and a shortage of specialized professionals. These differences provide a rationale for the formulation of differentiated regional policies.

### 4.4 International comparisons and implications

#### 4.4.1 International comparisons

China’s spatial evolution model for the supply of public fitness services shares both similarities and differences with those of other countries. Compared with developed countries, China has achieved a rapid increase in service supply levels and accelerated catch-up growth in less-developed regions within a short period, thanks to its strong central coordination and policy transmission mechanisms. Compared with developed countries in Europe and North America, China’s regional disparity may be more pronounced. For instance, the United States has enacted legislation such as the Leisure and Health Act to ensure the equitable allocation of community sports facilities, while the European Union provides special subsidies to less-developed regions through its Cohesion Policy. These international experiences suggest that relying solely on local autonomous development or market mechanisms is insufficient to automatically achieve spatial equity in fitness services; higher-level institutional arrangements and cross-regional coordination mechanisms are necessary. China’s successful experience in narrowing the east–west gap—such as fiscal transfers and paired assistance mechanisms—can serve as a reference for other developing countries. Meanwhile, the widening north–south gap serves as a warning to emerging economies to remain vigilant against the emergence of “novel regional differentiation.”

#### 4.4.2 Implications for regional governance

First, consolidate the gains made in narrowing the east–west gap. It is recommended to continue increasing central government fiscal transfers to western provinces and to promote the replication and diffusion of mature fitness service models from the eastern region—such as smart sports platforms and government-purchased services—to the western region. Second, address the challenge of the widening north–south gap. Northern provinces should increase the supply of indoor fitness facilities (e.g., community fitness centers) to mitigate the constraints of cold winter weather on outdoor activities. At the same time, industrial support policies should be implemented to curb population outflows and stabilize the social foundation of service supply. Third, leverage the spillover effects of high-supply provinces. It is recommended to systematize the successful experiences of provinces such as Jiangsu, Zhejiang, and Guangdong into a replicable policy toolkit, which can be disseminated to low-supply provinces through cross-regional cooperation platforms. Meanwhile, targeted measures should be implemented to address the structural shortcomings of provinces such as Qinghai, Ningxia, and Xinjiang. Fourth, narrow the “digital divide” exposed by the pandemic. It is recommended to increase investment in digital fitness infrastructure in central and western provinces and to develop age-friendly fitness applications, ensuring that the benefits of digital transformation reach all residents.

## 5 Spatial spillover effects of the supply level of public services for national fitness

### 5.1 Spatial correlation test

Table 4 presents the results of the Moran’s I index analysis for the supply level of public services for national fitness from 2013 to 2022. As shown in the table, the Moran’s I values throughout this period were consistently greater than zero and passed the significance test in all years except 2014. This indicates that the spatial distribution of the supply level is not random but exhibits significant spatial agglomeration characteristics, thereby validating the appropriateness of employing a spatial econometric model (32). Furthermore, the Moran’s *I* values fluctuated across different years, reflecting an uneven intensity of spatial agglomeration over time and further suggesting that the spatial pattern of the supply of public services for national fitness still requires optimization.

**Table 4:** Results of Moran’s *I* index analysis.

Year	Moran’s *I*	*p*-value	Year	Moran’s *I*	*p*-value
2013	0.158	0.052	2018	0.123	0.093
2014	0.100	0.130	2019	0.149	0.062
2015	0.153	0.060	2020	0.212	0.019
2016	0.191	0.029	2021	0.209	0.020
2017	0.190	0.031	2022	0.231	0.012

### 5.2 Spatial econometric model selection

Table 5 reports the results of spatial econometric model tests. A variance inflation factor (VIF) test was conducted to diagnose multicollinearity among the influencing factors, with all VIF values falling below the threshold of 5, indicating that no serious multicollinearity issues exist in the model (33). Regarding model specification, the optimal model form was determined through a systematic testing procedure following the spatial econometric model decision-making framework (34). First, the Lagrange Multiplier (LM) test for spatial dependence was conducted. Both the LM-Error and LM-Lag tests, along with their robust forms, were statistically significant, rejecting the null hypothesis of “no spatial autocorrelation.” This indicates that a spatial econometric model should be employed rather than traditional OLS regression. Among these, the Robust LM-lag test exhibited a higher test statistic and significance level than the Robust LM-Error test, preliminarily favoring the Spatial Autoregressive (SAR) model. Second, the Hausman test was performed. The test result was significant (39.310, *p* < 0.01), rejecting the null hypothesis that the random effects model is consistent and efficient; therefore, the fixed effects model was selected. Third, the Likelihood Ratio (LR) test was used to determine the specific form of fixed effects. Both the LR-ind (27.710, *p* < 0.01) and LR-time (637.910, *p* < 0.01) tests were significant, rejecting the null hypotheses of “no individual effects” and “no time effects,” respectively, thereby supporting the use of a two-way fixed effects model with both individual and time effects. On this basis, the Wald test was conducted to assess whether the Spatial Durbin Model (SDM) could be simplified to the SAR or the Spatial Error Model (SEM). The test results (Wald-sar = 9.960, *p* = 0.126; Wald-sem = 5.620, *p* = 0.468) were both non-significant, indicating that the null hypotheses could not be rejected, and thus the SDM can be simplified to the more parsimonious SAR or SEM models. Following the principle of model parsimony commonly adopted in econometric analysis, the SDM was not selected. Combined with the higher significance of the SAR model suggested by the earlier LM tests, the SAR model was preliminarily selected as the estimation model for this study. In addition, the estimation results of the SEM are also reported (see Table 6) as a robustness check, and these results further support the rationale for selecting the SAR model.

**Table 5:** Test results for spatial econometric model selection.

Test method	Test indicator		Chi-square statistic		*p*-value	
LM test	LM Error		4.013		0.045	
	Robust LM-Error		22.474		0.000	
	LM-Lag		6.867		0.009	
	Robust LM-lag		25.328		0.000	
Fixed effects test	Hausman test		39.310		0.000	
LR test	LR-ind		27.710		0.002	
	LR-time		637.910		0.000	
Wald test	Wald-sar		9.960		0.126	
	Wald-sem		5.620		0.468	
Collinearity diagnostics	x1	x2	x3	x4	x5	x6
	4.220	2.860	1.120	2.310	1.930	4.820

**Table 6:** Spatial panel data regression results.

Variable	SAR model	SEM model
*ρ*	0.380*** (0.109)	
*λ*		0.241 (0.202)
x1	0.303*** (0.065)	0.453*** (0.048)
x2	2.874*** (1.085)	2.666** (1.112)
x3	0.035* (0.020)	0.035* (0.021)
x4	0.087* (0.047)	0.141** (0.007)
x5	0.102 (0.109)	1.105 (0.111)
x6	0.240** (0.118)	0.475*** (0.113)
Log-L	23.421	18.542
*R* ^2^	0.222	0.216

***, **, and * denote significance at the 1, 5, and 10% levels, respectively. Standard errors are reported in parentheses.

### 5.3 Spatial effect analysis

Table 6 reports the estimation results of both the SAR and SEM models under the economic-geographic weight matrix using a two-way fixed effects specification. The spatial autoregressive coefficient *ρ* of the SAR model is statistically significant at the 1% level, whereas the spatial error coefficient *λ* of the SEM model fails the significance test. Moreover, the SAR model yields a higher log-likelihood (Log-L) value and goodness-of-fit (*R*^2^) compared to the SEM model, indicating its superior explanatory power in the context of this study. This conclusion is consistent with the preceding model selection test results. Accordingly, the SAR model with two-way fixed effects is selected for subsequent analysis.

According to the SAR model estimation results reported in Table 6, the spatial autoregressive coefficient *ρ* is significantly positive at the 1% level, indicating a pronounced spatial dependence in the supply level of public services for national fitness across Chinese regions, whereby a positive spatial spillover effect exists between neighboring areas. From the perspective of transmission mechanisms, this positive spillover is realized primarily through three channels: first, policy diffusion, whereby successful policy experiences from neighboring provinces (e.g., government service procurement models, smart sports platform development) are readily learned and imitated by local governments; second, resource mobility, whereby capital, talent, and sports event resources flow across provinces, benefiting surrounding areas; and third, regional competition, whereby local governments engage in yardstick competition in the public service sector, and improvements in neighboring provinces’ supply levels pressure local governments to follow suit. These mechanisms collectively constitute the transmission network of inter-provincial spatial spillovers. Regarding the control variables, the levels of economic development, population density, government support, population educational structure, and urbanization all demonstrate significant positive effects on the supply of public services for national fitness. In contrast, although the coefficient for transportation convenience is positive, it fails the significance test, suggesting a potential promotional effect that lacks sufficient statistical evidence. As a robustness check, the coefficient signs and significance levels of all variables in the SEM model are largely consistent with those in the SAR model, confirming the robustness of the findings reported above.

### 5.4 Spatial effect decomposition

To further elucidate the spatial mechanisms through which various factors influence the supply level of public services for national fitness, this study decomposes the effects of the SAR model, with the results presented in Table 7. The effect decomposition comprises direct effects, indirect effects, and total effects. The direct effect captures the local impact of a given factor on the supply level within the region itself; the indirect effect characterizes the cross-regional influence exerted on neighboring areas through spatial linkages; and the total effect represents the overall impact of the factor on the entire region. To enhance the robustness of the research findings, spatial regressions were conducted using both a geographic distance matrix and a contiguity matrix to test the sensitivity of the results to alternative spatial weight specifications.

**Table 7:** Decomposition results of spatial spillover effects.

Variable	Economic-geographic weight matrix			Geographic distance weight matrix			Contiguity matrix		
	Direct effect	Indirect effect	Total effect	Direct effect	Indirect effect	Total effect	Direct effect	Indirect effect	Total effect
x1	0.307*** (0.064)	0.181*** (0.059)	0.487*** (0.060)	0.301*** (0.063)	0.192*** (0.062)	0.494*** (0.062)	0.392*** (0.051)	0.091*** (0.032)	0.483*** (0.047)
x2	2.804*** (0.952)	1.882 (0.362)	4.686** (2.137)	2.833*** (0.954)	2.055 (1.483)	4.888** (2.253)	2.435** (0.947)	0.608 (0.405)	3.043** (1.282)
x3	0.037* (0.021)	0.023 (0.018)	0.060* (0.036)	0.037* (0.021)	0.024 (0.019)	0.061* (0.037)	0.033 (0.022)	0.007 (0.006)	0.040 (0.026)
x4	0.092* (0.050)	0.059 (0.048)	0.151* (0.089)	0.090* (0.050)	0.062 (0.051)	0.152 (0.092)	0.074 (0.050)	0.017 (0.015)	0.091 (0.062)
x5	0.084 (0.128)	0.057 (0.098)	0.141 (0.219)	0.087 (0.128)	0.064 (0.106)	0.151 (0.227)	0.130 (0.130)	0.034 (0.040)	0.164 (0.166)
x6	0.252** (0.125)	0.135** (0.063)	0.387** (0.166)	0.239* (0.125)	0.137** (0.068)	0.376** (0.173)	0.406*** (0.102)	0.093** (0.037)	0.499*** (0.113)

***, **, and * denote significance at the 1, 5, and 10% levels, respectively. Standard errors are reported in parentheses.

As shown in Table 7, both the direct and indirect effects of economic development and urbanization levels are significantly positive, indicating that they not only directly enhance the local supply level of public fitness services but also generate positive radiation and spillover effects on surrounding regions. The direct effect reflects the promotional impact of local economic development and urbanization on the local supply of fitness services. Economically developed regions typically have more robust fiscal security systems and more dynamic market environments, enabling them to continuously increase investment in national fitness infrastructure and public services. Meanwhile, improvements in urbanization levels help optimize the efficiency of public resource allocation and enhance service accessibility and coverage, thereby directly improving local service supply capacity. The indirect effect reflects the radiation and driving role of local economic development and urbanization on the supply level of fitness services in neighboring regions. This enhancement effect, driven jointly by supply capacity and resource allocation, not only creates a virtuous cycle locally but also generates positive radiation to surrounding areas through demonstration effects, institutional imitation, and resource linkage. This spatially explains how economic development and urbanization contribute to the overall improvement in the regional supply level of public fitness services.

The direct effects of population density, government support, and educational structure are all significantly positive, but their indirect effects fail to pass the significance test. This finding reveals that the current spatial mechanism exhibits a pronounced “border effect”: the positive impacts of population agglomeration, government investment, and educational development are largely confined within provincial boundaries, making it difficult to generate effective spillovers across administrative borders. Mechanistically, the non-significant indirect effect of population density can be attributed to the geographic immobility of public fitness facilities, whose service radius is largely limited to local areas. Although higher population density can reduce per capita facility costs through economies of scale, this cost advantage cannot be “exported” to neighboring provinces. The non-significant indirect effect of government support stems from China’s fiscal decentralization system and place-based governance model—local governments’ public investments are spatially targeted, with fiscal funds primarily used within their own jurisdictions, lacking incentives for cross-regional mobility. The non-significant indirect effect of educational structure arises because residents with higher educational attainment have stronger demand for and willingness to supervise fitness services, thereby pressing local governments to improve supply through a localized “demand-response” mechanism. However, this demand pressure mainly acts on local governments and can hardly be transmitted to neighboring provinces.

The direct, indirect, and total effects of transportation accessibility are all positive but not statistically significant, indicating that although transportation infrastructure has shown a positive role, the magnitude of its impact has not yet reached a statistically significant level. Possible reasons include the following. First, at the provincial level, China’s transportation infrastructure is already relatively well-developed, and the issue of “whether roads exist” has been largely resolved. The real constraints may lie in the convenience of the “last mile” and the affordability of travel costs for low-income groups. Second, the positive effect of transportation conditions on public service supply may be subject to a threshold effect. The current level of transportation development may not have yet surpassed the critical threshold required to drive systematic optimization of public service resource allocation, and its positive impact on fitness service supply remains in a phase of quantitative accumulation. Third, current transportation improvements are mainly reflected in enhancing the convenience of individual travel, which is still insufficient to drive substantial changes in the public service supply pattern. Achieving a qualitative leap from “convenient travel” to “service optimization” requires effective integration and coordinated development between the transportation system and public service supply at the levels of planning, investment, and operation. Fourth, the use of provincial-level data in this study may mask urban–rural differences. Urban areas have relatively convenient transportation, while rural areas have weaker transportation accessibility; during the aggregation process, the positive and negative effects may cancel each other out, leading to statistically non-significant results.

Under different spatial weight matrix specifications—including the economic-geographic distance matrix, the geographic distance matrix, and the contiguity matrix—the regression results of the influencing factors on the supply level of public fitness services remain largely consistent. This indicates that the findings of this study possess good robustness and explanatory power (35) and do not undergo substantial changes due to the specific form of spatial connectivity assumed.

### 5.5 Robustness checks

To ensure the reliability of the research findings and to avoid estimation bias that may arise from specific choices in evaluation methods or indicator processing approaches, this study conducted robustness checks by substituting the standardization method. In the main regression, this study employed the CRITIC method for comprehensive evaluation, in which the standardization step used Min-Max normalization to transform all indicators to the [0, 1] interval. Although Min-Max normalization is a classical setting in the CRITIC method, its results may be susceptible to disturbance from extreme values in the indicators. To examine whether the evaluation results and spatial regression conclusions depend on a specific standardization method, this study further applied *Z*-score standardization to the raw indicator data, re-calculated the composite scores for each province based on the standardized data, and then re-estimated the SAR model. The results, as shown in column (1) of Table 8, indicate that the estimated coefficients of all control variables are largely consistent with those of the main regression in terms of both direction and significance, suggesting that the core conclusions of this study are reasonably robust.

**Table 8:** Robustness check results.

Variable	(1)	(2)	(3)	(4)
x1	0.321*** (0.0207)	0.293** (0.130)	0.309*** (0.0930)	0.251*** (0.0639)
x2	0.234*** (0.0163)	4.165*** (1.070)	5.504*** (1.504)	3.166** (1.267)
x3	0.0187 (0.0254)	0.0396* (0.0203)	0.0288 (0.0257)	0.0403* (0.0223)
x4	0.0714* (0.0403)	0.115** (0.0475)	0.0953* (0.0573)	0.0724 (0.0506)
x5	0.0318 (0.0215)	0.119 (0.112)	−0.0031 (0.141)	0.0913 (0.123)
x6	0.404*** (0.0303)	0.150 (0.114)	0.589*** (0.161)	0.200 (0.128)
*R* ^2^	0.401	0.208	0.071	0.223

***, **, and * denote significance at the 1, 5, and 10% levels, respectively. Standard errors are reported in parentheses.

To eliminate the potential interference of price factors on the estimation results, this study used 2013 as the base year and applied the corresponding price indices to deflate the x1 variable. The results are presented in column (2) of Table 8. The results show that after removing the price effects, the coefficient directions of all influencing factors remain consistent with those of the main regression. Although the significance levels exhibit minor fluctuations, they do not change substantially overall. This indicates that price factors have limited interference with the core conclusions, and the findings are reasonably robust. To further enhance the robustness of our conclusions, minimize human interference, and mitigate the potential disturbance of extreme values on the CRITIC objective weighting, the following procedures were adopted in the robustness checks: First, we deleted observations with missing values (i.e., when a specific indicator for a given province and year was missing, we removed that province–year observation), and applied annual winsorization at the 1st and 99th percentiles to all continuous indicators. On this basis, we recalculated the composite scores of the supply level of public fitness services for each province. Since spatial econometric models require balanced panel data, and to ensure that each year contains complete records for all 30 provinces, we further excluded years with missing data, ultimately retaining seven complete years—2014, 2016–2018, and 2020–2022—and re-estimated the SAR model. The results, as shown in column (3) of Table 8, indicate that, with the exception of a change in the coefficient direction of x5, the coefficient directions and significance levels of the other variables remain largely consistent with those of the main regression. The absolute coefficient of x5 is small, and it was not statistically significant in the main regression either, suggesting that this variable has a relatively limited impact on the supply level of public fitness services, and the change in its coefficient direction does not materially affect the core conclusions. Overall, the robustness check results are broadly consistent with those of the main regression, indicating that the core conclusions of this study are reasonably robust and are not substantially affected by the data cleaning procedures or extreme value treatment methods.

In addition, the issue of endogeneity among indicators should also be taken into consideration. Theoretically, economic development level and urbanization level are the most likely variables to have a bidirectional causal relationship with the supply level of public fitness services: economically developed regions with higher urbanization levels possess more abundant fiscal resources and better infrastructure, which can enhance the supply level of public fitness services; conversely, improvements in the supply level of public fitness services can help improve residents’ health and human capital, thereby feeding back into regional economic growth and further promoting urbanization. In contrast, variables such as population density, government support, educational structure, and transportation accessibility are less likely to be affected by reverse causality—government support, as an indicator of fiscal expenditure structure, follows a decision logic whereby the government actively allocates resources rather than passively responding to changes in fitness service levels; educational structure is the result of long-term historical accumulation and is not affected by fitness service supply in the short term; transportation accessibility depends on regional infrastructure planning, and the reverse causality logic does not hold; population density is primarily determined by historical, geographical, and economic factors. Therefore, the potential endogeneity risk in this study is mainly concentrated on the two variables of economic development and urbanization. To mitigate this bias, this study re-estimated the SAR model using one-period lags of economic development level and urbanization level. If reverse causality indeed exists, contemporaneous explanatory variables would be influenced by the dependent variable, whereas one-period lagged explanatory variables would not be affected by the current dependent variable. The robustness check results [see column (4) of Table 8] show that the coefficient direction and significance level of x1 are generally consistent with those in Table 6; the coefficient directions and significance levels of x2, x3, and x5 remain largely unchanged; while the coefficient directions of x4 and x6 remain consistent, their significance declines. The possible reasons are that the impact of urbanization level on fitness service supply may have a certain lag effect—the achievements of current urbanization construction often take time to translate into fitness services accessible to residents, so when the current value is replaced by its one-period lag, the explanatory power of urbanization on current supply naturally weakens. Educational structure is a highly stable long-term structural variable with little annual variation, and the lagged treatment further reduces its variability, leading to a decline in significance. In addition, the lagged treatment shortens the sample period from 2013 to 2022 to 2014–2022, and the reduction in sample size may also have a certain impact on estimation efficiency. In summary, although the significance of some variables changes in the lagged model, the coefficient directions of all influencing factors remain consistent with those of the baseline model, and the estimation results for most variables are reasonably robust. This suggests that reverse causality poses only limited interference to the main conclusions of this study. It should be noted that the coefficients reported in this study primarily reflect statistical associations and spatial dependence patterns among variables, rather than strict causal relationships. Future research may employ instrumental variable approaches or quasi-experimental designs to more rigorously identify causal mechanisms.

### 5.6 International comparisons and implications

#### 5.6.1 International comparisons

The findings on spatial spillover effects in this study share both commonalities and differences with the international literature. In terms of commonalities, the positive spatial spillover effects of economic development and urbanization in this study are consistent with findings from developed countries. Policy innovation and resource allocation models in economically developed regions typically generate radiation and driving effects on surrounding areas, a phenomenon widely documented in regional development studies in Europe and North America (e.g., research related to the EU Cohesion Policy). In terms of differences, European and North American countries rely mainly on population migration and fiscal transfer mechanisms. From the perspective of population mobility, inter-provincial labor migration can affect the supply level of fitness services through two channels: first, migrants bring fitness demands from their places of origin to their destinations, stimulating diversification of service supply; second, return migrants bring advanced service concepts back to their places of origin, promoting the renewal of service models in less-developed regions. Due to data availability limitations, this study was unable to precisely quantify these effects. From the perspective of policy transfer, knowledge flow and institutional learning are important transmission mechanisms of spillover effects. Innovative practices in developed regions (such as smart sports platforms and government-purchased services) are learned and imitated by less-developed regions through channels such as official visits and experience exchanges, a process moderated by factors such as geographical distance, administrative hierarchy, and performance pressure. The mutual reinforcement between population mobility and policy transfer jointly drives the fitness service supply model to break through the constraints of provincial administrative boundaries through the dual cross-border flow of “people” and “information,” achieving regional diffusion and convergence. The uniqueness of inter-provincial spillovers in China is reflected in the following two points. First, the dominant role of policy diffusion and regional competition is more prominent. Unlike European and North American countries, inter-provincial spillovers in China rely more on policy transmission within the administrative system and yardstick competition among local governments. The finding that population density has no significant indirect effect in this study is consistent with this judgment—factors such as the household registration system limit the role of population migration in spillovers. Second, the hindering effect of administrative boundaries on spillovers is more pronounced. The finding that variables such as government support and educational structure have “direct effects but no spillover effects” in this study reflects that, under China’s fiscal decentralization system and place-based governance model, the spatial spillover of public investment is significantly constrained by administrative boundaries. In contrast, the European Union has, through institutional arrangements such as cross-border cooperation zones and structural funds, weakened the fragmenting effect of national borders on public services to some extent.

The above comparison indicates that China has both strong advantages in policy transmission and challenges posed by administrative fragmentation. International experience suggests that establishing cross-regional coordination mechanisms and horizontal transfer payments is an effective path to break through administrative boundaries and unlock the potential of spatial spillovers.

#### 5.6.2 Implications for regional governance

The findings on spillover effects in this study have important implications for the coordination of public policies at the regional level. First, unleash the positive spillovers of economic development and urbanization. It is recommended to promote the establishment of cross-regional public service collaboration platforms to facilitate resource sharing, joint facility construction, and mutual exchange of experiences. Priority can be given to exploring integrated supply models of fitness services within city clusters such as the Yangtze River Delta, Pearl River Delta, and Beijing-Tianjin-Hebei region, and then promoting replicable experiences nationwide. Second, break through the constraints of administrative boundaries on spillovers. It is recommended to establish cross-provincial horizontal transfer payment and collaboration mechanisms, whereby net beneficiary regions of spillover effects compensate net adversely affected regions. For example, developed eastern provinces can support central and western provinces in enhancing their fitness service supply capacity through financial assistance, talent exchange, and technology transfer. Third, re-examine the role of transportation infrastructure in fitness services. The finding that transportation accessibility is not significant suggests that simply pursuing an increase in road network density is insufficient to automatically improve fitness service supply. Future efforts should focus on “last-mile” supporting infrastructure, promote the coordination between transportation planning and fitness facility layout, reduce travel costs for low-income groups, and achieve a qualitative leap from “convenient travel” to “service optimization.”

## 6 Conclusion and discussion

### 6.1 Conclusion

Using panel data from 30 Chinese provinces spanning 2013 to 2022, this study systematically measured the supply level of nationwide fitness public services using the CRITIC method, and examined its spatiotemporal evolution characteristics and spatial spillover effects through spatial trend surface analysis, spatial autocorrelation tests, and a spatial lag model. The main conclusions are as follows:

First, from 2013 to 2022, the supply level of nationwide fitness public services in China exhibited a steady three-stage upward trend, with an average annual growth rate of 7.18%. This development process can be divided into a rapid expansion stage (2013–2017), a dynamic adjustment stage (2018–2020), and a resilient quality improvement stage (2021–2022). At the regional level, the supply level followed a gradient pattern of Eastern > Central > Northeastern > Western China, with persistent regional imbalances remaining prominent.

Second, the spatial pattern of nationwide fitness public service supply is characterized by higher levels in the east and south, and lower levels in the west and north. The east–west gap has gradually narrowed, reflecting the effectiveness of national coordinated regional development strategies, whereas the north–south gap has continued to widen, signaling emerging regional health disparities. High-supply provinces have gradually diffused from the eastern coastal areas toward the central, western, and northeastern regions, demonstrating the positive role of policy guidance and resource redistribution.

Third, spatial econometric analysis reveals a significant spatial dependence in the supply of nationwide fitness public services. Economic development level and urbanization level exert significant bidirectional positive spatial spillover effects, benefiting both local and neighboring regions’ supply levels. In contrast, population density, government support, and population educational structure only promote local supply, with no significant cross-regional spillover effects. The impact of transportation accessibility on service supply is not statistically significant.

Overall, the findings confirm that the supply level of nationwide fitness public services in China has improved significantly over the past decade, yet spatial inequality and regional development imbalances persist. Socioeconomic factors, particularly economic development level and urbanization level, play a critical role in shaping the spatial pattern of service distribution and driving cross-regional spillover effects, whereas administrative boundaries and local resource constraints limit the diffusion of other factors. These findings provide empirical evidence for promoting health equity and advancing the equalization of nationwide fitness public services.

### 6.2 Discussion

The findings of this study make the following theoretical contributions to the literature on the social determinants of health, spatial justice, and public service governance. First, they validate and extend the applicability of the social determinants of health theory to the domain of fitness services. The significant effects of economic development, urbanization, and educational structure on the supply level of public fitness services align with the core proposition of the social determinants of health theory—that disparities in health outcomes are rooted in the territorial inequality of socioeconomic conditions. The finding that the east–west gap is narrowing while the north–south gap is widening suggests that policy interventions can reshape the spatial pattern of the social determinants of health and thereby promote health equity. Second, this study provides empirical evidence from China for spatial justice theory. The positive spatial spillover effects of economic development and urbanization identified in this study align with the ideal state of spatial justice. However, the finding that population density, government support, and educational structure exhibit significant direct effects but no spillover effects reveals the institutional barriers to spatial injustice. This indicates that relying solely on local autonomous development or market mechanisms is insufficient to automatically achieve spatial justice in public services; higher-level cross-regional coordination mechanisms are necessary. Third, this study highlights the added value of spatial methods in health equity research. Traditional studies have mostly employed non-spatial models that assume regions are independent of one another, failing to capture cross-regional spillover effects. By employing spatial econometric models, this study distinguishes between direct and indirect effects, revealing both diffusion effects and blocking effects.

From a public health perspective, this study proposes three key policy priorities. First, targeted regional policies should be formulated to address the widening north–south gap, including increased financial and technical support for northern provinces, with a focus on improving their fitness infrastructure, the allocation of professional instructors, and service network coverage, as well as the establishment of differentiated evaluation mechanisms. Second, intergovernmental collaborative governance should be strengthened to fully unleash the positive spatial spillover effects of economic development and urbanization. Improvements in economic and urbanization levels can not only drive local supply but also benefit neighboring regions. Therefore, efforts should be made to promote the establishment of cross-regional public service collaboration platforms, facilitating resource sharing, joint facility construction, and mutual exchange of experiences, thereby alleviating service disparities caused by administrative fragmentation. Third, resource allocation should be optimized with a focus on health equity, targeting grassroots and disadvantaged areas. Service supply should not merely pursue aggregate growth; priority should instead be given to rural, remote, and low-income areas to ensure that vulnerable populations have equal access to fitness facilities, health guidance, and physical fitness monitoring services. The concept of health equity should be embedded throughout the entire supply process. It should be noted that this study primarily employs the spatial autoregressive (SAR) model, which reveals statistical associations and spatial dependence patterns among variables rather than strict causal relationships. Although an endogeneity test using a one-period lagged model was conducted in Section 5.5, and the results showed that the estimates for economic development level are relatively robust, the potential biases caused by reverse causality and omitted variables cannot be completely ruled out. Therefore, the coefficients reported in this study should be interpreted as “spatial associations” rather than “causal effects,” and readers should exercise caution when drawing policy implications. Future research may employ instrumental variable approaches or quasi-experimental designs to more rigorously identify causal mechanisms.

This study has the following limitations. First, the analysis is based on provincial-level aggregate absolute data, which may not only mask inter-county and urban–rural disparities in service development within provinces, but also suffer from specific applicability boundaries, as the absolute indicators do not account for the influence of population size differences. Future research could employ data at finer spatial scales, such as prefecture-level or county-level data, and incorporate per capita standardized relative indicators to conduct multi-dimensional comparative analyses, thereby providing a more comprehensive and precise depiction of the regional disparities and spatial patterns in the supply level of public fitness services. Second, the absence of health outcome indicators. This study focuses on the level of service supply and does not directly link supply indicators to population health outcomes (e.g., physical activity rates, obesity rates, chronic disease prevalence, life expectancy, etc.). Third, insufficient incorporation of emerging factors. This study does not directly capture the impact of the rapid development of digital technologies—such as artificial intelligence, wearable devices, and online fitness platforms—on the spatial pattern of services. Fourth, limitations in causal identification. This study primarily reports statistical associations and spatial dependence patterns and does not fully address endogeneity concerns. Future research may delve deeper in the following directions. First, adopt finer spatial scales. Future studies could employ prefecture-level or county-level data to reveal more detailed spatial patterns and identify the extent of “within-province inequality” and its implications for health equity. Second, link to health outcome indicators. Future research could match public service data with large-scale health survey data (e.g., the China Health and Nutrition Survey) to quantify the marginal contribution of fitness service supply to health indicators such as life expectancy, obesity rates, and chronic disease prevalence. Third, explore the spatial effects of digitalization. Future studies could investigate whether digital transformation narrows or widens regional disparities in fitness services, and how digital platforms can be leveraged to enhance service accessibility in less-developed regions. Fourth, pay attention to urban–rural differences. Future research should separately examine the patterns of fitness service supply in urban and rural areas, identifying the differences between the two types of regions in terms of resource allocation, service accessibility, and health equity, as well as the mechanisms underlying these differences.

## Data Availability

The original contributions presented in the study are included in the article/supplementary material, further inquiries can be directed to the corresponding author.

## References

[1] Central Committee of the Communist Party of China Resolution on Further Deepening reform Comprehensively to Advance Chinese Modernization. Chinese Government Online. (2024). Available online at: https://www.gov.cn/zhengce/202407/content_6963770.htm (accessed September 6, 2025).

[2] Mass Sports Department, General Administration of Sport of China Key work Priorities for mass Sports in 2026. Official Website of the General Administration of Sport of China. (2026). Available online at: https://www.sport.gov.cn/qts/n4986/c29448608/content.html (accessed February 29, 2026).

[3] Xu P Zhu L Yang L Yan J. Manpower, financial, material resources, and participation level in national fitness: a fuzzy-set QCA approach. Front Public Health. (2024) 12:1375930. doi: 10.3389/fpubh.2024.1375930, 39421810 PMC11484622

[4] Downward P Inoue Y Kumar H Widdop P. The locality challenges facing the ‘levelling up’of sport participation and health inequality in England. Int J Sport Policy Polit. (2025) 17:327–44. doi: 10.1080/19406940.2024.2404949

[5] Zheng J. Prospects and practical strategies for improving the public service system for national fitness during the “15th five-year plan” period. J Shanghai Univ Sport. (2026) 50:48–57. doi: 10.16099/j.sus.2025.08.18.0007

[6] Liu Z Zhang S Li L Hu B Liu R Zhao Z . Research on the construction and prediction of China's national fitness development index system under social reform. Front Public Health. (2022) 10:878515. doi: 10.3389/fpubh.2022.878515, 35651855 PMC9149160

[7] Jiang S Lu L Wang H Liu J Xu J Li Q. Research on the spatial allocation of national fitness resources at the street scale—taking Fuzhou city as an example. Heliyon. (2024) 10:e29293. doi: 10.1016/j.heliyon.2024.e29293, 38633626 PMC11021966

[8] Zheng J Luan L Sun M. Does the national fitness policy promote national health?—an empirical study from China. Int J Environ Res Public Health. (2022) 19:9191. doi: 10.3390/ijerph19159191, 35954550 PMC9368402

[9] Gao W Feng W Xu Q Lu S Cao K. Barriers associated with the public use of sports facilities in China: a qualitative study. BMC Public Health. (2022) 22:2112. doi: 10.1186/s12889-022-14441-w, 36401218 PMC9672562

[10] Ren P Liu Z. Efficiency evaluation of China’s public sports services: a three-stage DEA model. Int J Environ Res Public Health. (2021) 18:10597. doi: 10.3390/ijerph182010597, 34682343 PMC8535537

[11] Nessel K Kościółek S. The total sporting arms race: benchmarking the efficiency of public expenditure on sports in EU countries. Eur Sport Manag Q. (2022) 22:833–55. doi: 10.1080/16184742.2020.1833956

[12] Yu C Li T Liu X. How government investment influence the national fitness? SAGE Open. (2025) 15:21582440251382625. doi: 10.1177/21582440251382625

[13] Lan Q Liu C Ling S. Research on measurement of symbiosis degree between national fitness and the sports industry from the perspective of collaborative development. Int J Environ Res Public Health. (2019) 16:2191. doi: 10.3390/ijerph16122191, 31234272 PMC6617403

[14] Zhou M Wang X. AI-empowered supply of public services for national fitness: an analytical framework and implementation strategies. J Beijing Sport Univ. (2025) 48:33–49. doi: 10.19582/j.cnki.11-3785/g8.2025.04.003

[15] Chen X Wang Z Liu L Gao K. The operational model and generative logic of smart governance in promoting public sports service supply in urban communities: a multi-case comparative study. J Sport Res. (2025) 39:87–98. doi: 10.15877/j.cnki.nsic.20250408.002

[16] Dong X Yi F Wang Z. Informatization of national public service fitness in constructing a smart city using big data. Sci Program. (2022) 2022:1–10. doi: 10.1155/2022/9583803

[17] Li L Yang A. A recommendation method of national fitness items based on neural network algorithm. Sci Program. (2022) 2022:1–10. doi: 10.1155/2022/1544767

[18] Gordon-Larsen P Nelson M Page P Popkin B. Inequality in the built environment underlies key health disparities in physical activity and obesity. Pediatrics. (2006) 117:417–24. doi: 10.1542/peds.2005-0058, 16452361

[19] Dincă M Antohi V Andronic M Szeles M Baba C. Modelling health financing performance in Europe in the context of macroeconomic uncertainties. Economies. (2023) 11:299. doi: 10.3390/economies11120299

[20] Pricope L. Antohi V. Meca A. Buboi A. Fortea C. Zlati M. The new European development scoreboard for SDG11 at the European level. Sustainability (2024) 16:7736. do:doi: 10.3390/su16177736

[21] Yang B Hassan F Ahmad W Antohi V Fortea C Condratovici C. Impact of air pollution on life expectancy in Asian developing countries: does renewable energy adoption matter? Front Public Health. (2026) 14:24402. doi: 10.3389/fpubh.2026.1724402, 42007359 PMC13083176

[22] Bo T Lei Y. Regional differences and convergence of the supply level of public services for national fitness in China: from the perspective of higher-level public services for national fitness. J Chengdu Sport Univ. (2024) 50:34–41. doi: 10.15942/j.jcsu.2024.02.005

[23] Hu Q Song K Cao J Zhang D. Spatiotemporal differentiation, convergence characteristics, and evolutionary trends of the supply level of basic public services for national fitness. China Sport Sci. (2025) 45:25–37. doi: 10.16469/J.css.2025KX056

[24] Gao X Cao L Gu Q. Assessing the efficiency of China’s national fitness public services: a super-efficiency DEA-Malmquist–Tobit approach. Front Public Health. (2024) 12:1433337. doi: 10.3389/fpubh.2024.1433337, 39328996 PMC11424459

[25] Che L Bai Y Zhou L Wang F Ji X Qiao F. Spatial characteristics and spillover analysis of green development efficiency in China. Sci Geogr Sin. (2018) 38:1788–98. doi: 10.13249/j.cnki.sgs.2018.11.006

[26] Dong D He B Kong J Zhou A Lv M Zhang D . Can China’s national fitness policy contribute to achieving universal health? Analysis based on the three-dimensional framework. Front Public Health. (2025) 13:1610070. doi: 10.3389/fpubh.2025.1610070, 40900691 PMC12399634

[27] Sun H Cheng M Jiang F. Research on the influence mechanism of digital economy on the development of the sports and health industry in China. Sci Rep. (2025) 15:30595. doi: 10.1038/s41598-025-16641-x, 40835887 PMC12368095

[28] Cheng M Zhang L Zhang X Li Y. Does rural sports tourism promote the sustainable development of the destination?--based on quasi-experimental evidence of sports and leisure towns in China. J Clean Prod. (2025) 486:144537. doi: 10.1016/j.jclepro.2024.144537

[29] Zhang H He C Lu W. A study on the characteristics and influencing factors of public sports service demand among the population of rural older adults in China. Front Public Health. (2025) 13:1613449. doi: 10.3389/fpubh.2025.1613449, 40697852 PMC12279805

[30] Tao M Zheng J. Accessibility of public services for national fitness: conceptual connotation, value implications, and improvement pathways. J Chengdu Sport Univ. (2024) 50:42–49+84. doi: 10.15942/j.jcsu.2024.02.006

[31] Chen C Chen N. Building a higher-level public service system for national fitness: foundations, priorities, and practices. J Shanghai Univ Sport. (2024) 48:43–55+65. doi: 10.16099/j.sus.2024.01.04.0004

[32] Guo W Guo X Qiao H. Statistical measurement and spatiotemporal evolution characteristics of the sports industry development level. Stat Decis. (2025) 41:139–43. doi: 10.13546/j.cnki.tjyjc.2025.01.024

[33] Tao J Yu J. The spatial time lag in panel data models. Econ Lett. (2012) 117:544–7. doi: 10.1016/j.econlet.2012.07.025

[34] Elhorst J. Dynamic spatial panels: models, methods, and inferences. J Geogr Syst. (2012) 14:5–28. doi: 10.1007/s10109-011-0158-4

[35] Zhang Y Zheng R Wang Z. Regional evolution characteristics and driving factors of the integrated development level of the sports, culture, and tourism industry. J Xi'an Phys Educ Univ. (2025) 42:308–326+361. doi: 10.16063/j.cnki.issn1001-747x.2025.03.005

